# 轻舟已过万重山，直挂云帆济沧海祝贺《中国肺癌杂志》被Medline收录

**DOI:** 10.3779/j.issn.1009-3419.2010.08.01

**Published:** 2010-08-20

**Authors:** 清华 周

**Affiliations:** 300052 天津，天津医科大学总医院，天津市肺癌研究所，天津市肺癌转移与肿瘤微环境重点实验室 Tianjin Key Labortory of Lung Cancer Metastasis and Tumor Microenviroment, Tianjin Lung Cancer Institute, Tianjin Medical University General Hospital, Tianjin 300052, China

“百尺飞泉鸣震谷，一声长啸势惊天”。公元1998年7月，时值农历戊寅虎年，在各级领导的关怀下，在华西医科大学及华西医科大学附属第一医院的支持下，在我国著名肺癌专家、德高望重的孙燕院士的领导下，《中国肺癌杂志》创刊；而十二年后的今天——2010年7月，同为农历庚寅虎年，经美国National Institutes of Health机构咨询委员会（The Literature Selection Technical Review Committee, LSTRC）评定，《中国肺癌杂志》正式被Medline/Pubmed/Index Medicus收录！成为继《癌症》、《中华肿瘤杂志》后我国被Medline收录的第三本肿瘤学专业期刊，也是我国唯一一本被Medline收录的肿瘤专病学术期刊。值此《中国肺癌杂志》被Medline收录之际，我向本刊的第一任主编孙燕院士，向为本刊的创刊和发展作出过重要贡献的第一任编辑部主任张世雯女士、第一届编辑部工作人员李蓓兰、税艳玲女士，向现有编辑部的所有工作人员，尤其是向为本刊的发展、壮大作出最重要贡献的历届编委会的专家们以及广大作者和读者致以衷心的感谢和崇高的敬礼！向长期以来对本刊的发展给予大力支持的各级领导、本刊的主办单位的领导以及各级新闻主管部门的领导表示衷心的感谢和崇高的敬礼！正是得益于他们的支持、关心和帮助，我们才能梦想成真！

Pubmed系统是由美国国立生物技术信息中心（National Center for Biotechnology Information, NCBI）开发的用于检索Medline、PreMED-LINE数据库的网上检索系统。Medline是美国国立医学图书馆（U.S. National Library of Medicine, NLM）最重要的书目文摘数据库，内容涉及医学、护理学、牙科学、兽医学、卫生保健和基础医学。Medline创始于1966年，收录了全世界70多个国家和地区的5 000余种生物医学期刊，现有书目文摘条目1 000万余条。Index Medicus因提供世界生物医学文献的检索而广为应用，因此，各国的期刊主办者、出版商均希望自己的期刊被Medline收录。目前，全世界有生物医学刊物1.3万-1.4万种，所以NLM对刊物的选择非常严格。为保证收录期刊质量的可靠性，NLM于1988年成立了LSTRC，专门用于审核期刊、评价其内容，增选新刊，同时剔除不符合要求的已收录期刊。现该委员会由生物医学领域的医学家、科学家、教育家、编辑、卫生科学图书馆学家及医学历史学家等权威人士组成，这些专家对入选期刊进行严格评估，只有高质量生物医学期刊才可通过评审入选Medline。

最近五年以来，中文生物医学期刊每年入选Medline的数量仅为1-2本，而中文肿瘤期刊自2002年《癌症》被Medline收录以来便无其它同类期刊被收录。此次《中国肺癌杂志》被Medline/Pubmed数据库收录，是对本刊整体水平的肯定，也是对中国肺癌基础、临床研究水平的肯定，更是对中国广大肺癌防治工作者的基础和临床研究工作的认可，这无疑将为本刊和国内肺癌事业的进一步发展提供良好的契机。这既是一种荣誉，同时也是一份鼓励和鞭策。值《中国肺癌杂志》被Medline收录之际，我对本刊的办刊经验作一小结，并以此作为新的发展起点，再攀新高峰。

## 1 《中国肺癌杂志》的办刊特色

### 1.1 高起点办刊

在20世纪末，肺癌在我国的发病率和死亡率快速增长，对人群健康和生命构成极大威胁。与此同时，中国肺癌的研究快速发展，肺癌从业人员也大大增加，大批临床、科研人员投身肺癌事业，在肺癌基础研究和肺癌整合诊疗方面取得长足进步和重大成果。因此，迫切需要一本肺癌专业学术期刊，以加强我国肺癌的基础与临床研究，促进肺癌的学术交流，提高我国肺癌防治水平，造福大众。在此背景下，《中国肺癌杂志》在中国科学技术协会、中国抗癌协会、中国防痨协会和华西医科大学以及华西医科大学附属第一医院各领导的大力支持下于1998年7月创刊，并邀请到我国德高望重的著名肺癌专家孙燕院士担任首任主编。创刊之际，时任卫生部副部长的殷大奎教授拨冗撰写创刊祝词，全国人大副委员长吴阶平院士为本刊题写刊名，中华医学会副会长曹泽毅教授与吴孟超院士欣然题词。殷大奎教授在创刊祝词中鼓励本刊“应本着面向基础、面向世界、面向未来的原则，选择各类稿件。……希望《中国肺癌杂志》的创立能在我国肺癌基础研究和肺癌临床工作者之间架设一座桥梁，促进我国肺癌基础研究和临床工作者之间的学术交流，推动我国肺癌基础和临床研究的共同发展，以及肺癌基础研究成果尽早在临床应用”。创刊伊始，本刊即以国际化办刊视野，遵循国际ICMJE医学期刊标准（Uniform Requirements for Manuscripts Submitted to Biomedical Journals），提供文题、作者、机构、摘要、图表及参考文献的中英文对照，使所刊发文章易于被国际同行阅读引用。并按照国际标准进行编排、印刷和出版。

2007年，《中国肺癌杂志》编辑部由华西医院迁至天津医科大学总医院，在天津医科大学总医院领导的大力支持下，得以延续高水平办刊。

### 1.2 高水平的编委会

为实现高水平办刊，《中国肺癌杂志》组建了第一届编委会，涵盖肺癌基础与临床研究领域的各个学科、具有广泛的学术代表性的专家。这些编委专家均为本学科各个领域具有重要影响的专家，为《中国肺癌杂志》奠定了坚实的学术基础。至今，《中国肺癌杂志》历经四届编委会，逐步形成由170余位肺癌领域内具有广泛学科代表性及学术代表性、兼有国际肺癌学术权威的高水平专家组成的编委会。历届编委专家为本刊的发展倾注了极大的热情，积极宣传《中国肺癌杂志》并组稿、审稿，且提出大量建设性意见。正是由于得到编委专家的大力支持，《中国肺癌杂志》的学术水平才一直保持领先，并不断进步。

### 1.3 严格的稿件质量控制和高效的稿件处理流程

《中国肺癌杂志》创刊伊始，便严格遵循国际医学期刊编辑条例，所有稿件严格经过两位以上审稿人审阅并由主编/执行主编亲自定稿，坚决反对各种商业因素影响学术，力图杜绝各种学术不端现象的产生。严格的学术评审保证了本刊的高学术水平，2005年《中国肺癌杂志》刊发的王金万等作者的文章先后于2007年、2008年两次被列为科技部中信所公布的“中国百篇最具影响国内学术论文”便是例证。更有较多文章为国际重要学术期刊，如*Journal of Clinical Oncology*、*Journal of Thoracic Oncology*、*Lung Cancer、Proteomics*、*American Journal of Epidemiology*、*Cancer Biology and Therapy*、*Biochemical and Biophysical Research Communications*、*Biochimica et Biophysica Acta*、*European Journal of Pharmacology*等等引用，显示了本刊文章的学术价值。

随着网络时代和信息时代的来临，广大的临床、科研工作者越来越要求高效、专业的网络同行评议。为此，《中国肺癌杂志》自2009年斥资购入汤森路透集团旗下国际流行的著名稿件在线评审系统Scholarone Manuscripts（http://mc03.manuscriptcentral.com/cjlc），真正实现与国际学术期刊同步。通过应用Scholarone Manuscripts系统，对稿件进行严格评审，英文稿件实行国际化评审，稿件评审周期控制在一个月之内，发表周期平均在四个月之内，深受广大作者和审稿专家的欢迎。

### 1.4 学术内容的时效性与先进性

为提高《中国肺癌杂志》学术内容的先进性，及时准确反映国内外肺癌领域最新临床、科研动态，广大编委专家做了大量工作，撰写了许多立题新颖、内容丰富的述评、综述，并组织出版了系列学术热点专题/专辑。《中国肺癌杂志》出版专题/专辑全面报道了世界肺癌大会、中国肺癌学术大会、中国肿瘤转移大会等学术会议，反映国内外肺癌基础与临床进展；并且刊发了“肺癌分子生物学”、“端粒酶研究”、“肺癌放射治疗”、“肺癌转移”、“肺癌化疗”、“肺癌多学科治疗”、“肺癌外科治疗”、“N2期肺癌”、“EBUS与肺癌”等等系列专题，提高了期刊的先进性。2009年，《中国肺癌杂志》刊期由双月刊变更为月刊，缩短了期刊的刊发周期，大大提高了文章的时效性，为广大作者和读者提供了更高质量的服务。

### 1.5 栏目设置丰富，注重提高内容的可读性

为全面反映肺癌从业人员各学科、领域的学术进展，并兼顾基础与临床，《中国肺癌杂志》栏目设置丰富，包括临床指南、述评、基础研究、临床研究、临床经验、技术进展、护理园地、综述、短篇报道、病例报道、会议进展等等。为反映最新国际研究进展，本刊还设有翻译类栏目“期刊博览”，刊发得到版权授予的翻译类精品文章。这些栏目反映了不同层次的基础和临床进展，文章题材丰富，信息量大。同时，《中国肺癌杂志》每期刊出大量会议信息、学术消息、书讯，并有最新国际期刊与协会彩版学术广告信息，向读者介绍最新和最权威的学术活动，大大提高了每期杂志的信息量。目前，《中国肺癌杂志》与美国临床肿瘤学会ASCO、国际肺癌研究会IASLC、韩国肺癌研究会KASLC、台湾肺癌学会、美国Wiley-Blackwell、Lippincott、Williams & Wilkins、Clinical Lung Cancer、Future Science、Landes Bioscience、BioMed Central等学术/出版团体建立稳定的交流合作，将为广大读者提供更新、更实用的学术新知。

### 1.6 先进的网络化平台与广泛的学术展示度

现代学术出版早已进入网络化时代，权威学术期刊往往可以将最新、最权威的学术内容通过网络向广大读者迅捷呈现。为实现符合国际标准的在线全文平台，本刊从2008年开始应用加拿大Open Journal System（OJS）系统建设电子期刊，并成功申请到顶级域名（www.lungca.org）和电子期刊刊号（eISSN 1999-6187），实现了全文HTML与PDF两种格式全文发布。OJS系统是开源多语言系统，具有丰富的扩展性，非常适合建立Open Access（OA）期刊。《中国肺癌杂志》在线电子期刊已经逐步建成符合国际标准的OA期刊，并被世界著名的OA期刊数据库DOAJ收录，成为其中少数中文OA期刊之一（图 1）。同样在2008年，本刊采用了数字对象唯一标识符（Digital Object Unique Identifier, DOI），使每篇文章获得了国际认可的身份标识。为提高学术内容的时效性，《中国肺癌杂志》实现了当期电子全文与印刷版同时出版，极大地提高了期刊展示度。随着本刊学术水平不断被国内外所认可，《中国肺癌杂志》陆续被科技部科技核心期刊目录、EMBASE/SCOPUS、CA、CINAHL、CAB Abstract、Global Health、Index Copernicus、Medline/Pubmed等等检索系统收录。最重要的是，《中国肺癌杂志》电子版文章通过与Pubmed的全文链接，将使当期发表的文章在较短的时间内即可被国际同行检索、下载阅读，极大地提高了本刊文章的学术影响力和国际展示度（图 2）。

**1:**
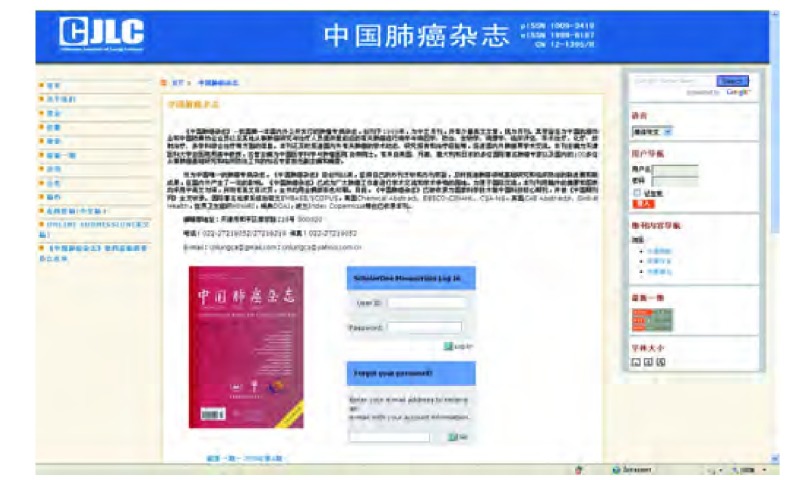
《中国肺癌杂志》应用OJS建设全文网站截图 Snapshot for OA fulltext linkout website for *Chinese Journal of Lung Cancer* established with Open Journal System (OJS)

**2:**
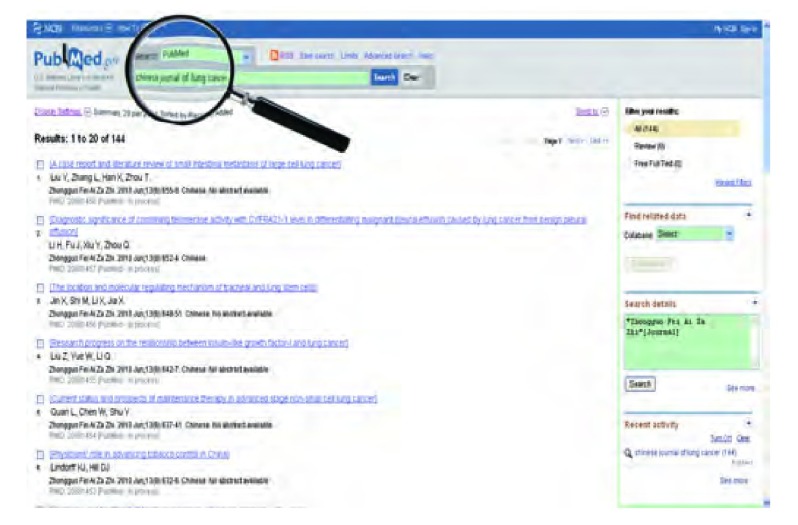
《中国肺癌杂志》自Pubmed全文链接 Fulltext linkout from Pubmed to *Chinese Journal of Lung Cancer*

### 1.7 加强学会联系，服务会员

《中国肺癌杂志》为中国抗癌协会肺癌专委会官方刊物，因此为学会会员服务是本刊的宗旨之一。《中国肺癌杂志》现在已经实现向所有肺癌专委会会员免费寄送当期刊物，并免除所有审稿费用，实现免费开放全文。本刊积极支持学会的各项学术活动，听取会员意见与建议，并面向广大会员开展继续医学教育、赠刊、学术征文等活动，取得良好反响。

### 1.8 国际化期刊编排与装帧

学术期刊的编排与装帧是期刊的外在表现，一定程度反映了期刊的办刊水平与编者学术态度。为缩短与国内外同行的差距，本刊自创刊，便学习和借鉴了大量国内外优秀学术期刊的办刊经验和管理流程，按照国际标准设立期刊编校规范，特别是文题、署名、单位、摘要及参考文献均要求中英文对照，便于与国际同行交流并了解中国的研究成果。为适应国际电子出版的潮流，《中国肺癌杂志》排版软件逐渐由方正书版变更为Adobe Indesign，后者可以生成标准Adobe PDF文件，与Adobe系列出版软件兼容，易于实现网络化与电子出版。对纸本期刊的装帧与印刷，编辑部一直以高水准要求自己，尽力将最完美的期刊呈现给读者。

## 2 存在的问题与展望

尽管《中国肺癌杂志》已取得一定的成果，但是与国内外同行相比仍有较多不足。

### 2.1 高质量稿件不足，学术水平仍待提高

《中国肺癌杂志》所刊发的大规模随机双盲临床文章较少，创新性高的基础研究文章不多，有重要影响力的文章有限。在目前国内的科研评价体系下，国内高水平的研究成果往往发表在国际英文SCI期刊上，重要的基础、临床科研成果发表在中文刊上的较少。究其原因，除科研评价体系外，语言问题也是重要方面，因为英文文章往往容易被国际同行阅读、引用。中文医学期刊必须要通过提高服务水平，贴近基础和临床科研人员，学习最新学科进展，才可能获得高质量稿件。

另外，随着国际影响力的增大，本刊在过去多年中陆续收到国内外英文稿件，其中不乏高质量的文章。为向世界展示亚太地区肺癌研究的最新进展，我们联合国际华人胸外科协会和韩国相关肺癌组织创办了英文刊*Thoracic Cancer*（www.thoraciccancer.net），由国际著名出版社Wiley-Blackwell出版，希望将该刊办成具有一定影响力的胸部肿瘤学国际期刊（图 3）。我们也欢迎国内广大肺癌、食管癌、纵隔肿瘤等胸部肿瘤从业人员积极赐稿、阅读文章。

**3:**
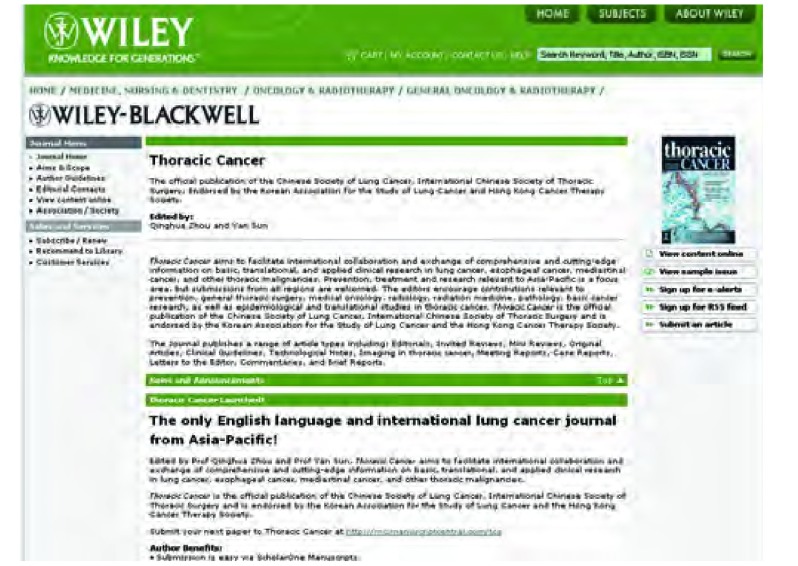
英文刊*Thoracic Cancer*在Willy-Blackwell的主页 Homepage of *Thoracic* *Cancer* published with Willy-Blackwell

### 2.2 期刊影响力仍有待提高

《中国肺癌杂志》创刊至今仅有十二年，而国内肿瘤及胸外科期刊有数十本，其中不乏创刊久、影响力大的医学期刊。这些期刊学术认可度高，读者群庞大，具有较高的影响力。我们应该努力向这些高水平期刊学习，结合现代化办刊手段，不断提高科研服务水平。国际先进医学期刊的办刊模式将陆续被本刊采用，例如提前在线出版（Epub ahead of print），接受稿件的终稿在纸本发表之前可以提前在本刊网站电子发表，并能被Pubmed检索并全文链接，这将大大提高文献的时效性和被阅读机率，具有重要的学术意义；特色文章（Featured article）的选择，即选出有重要学术价值的文章在封面展示文题，并邀请专家作出点评，免除相关稿件所有发表费用，并获得绿色通道快速发表的机会（2个月内刊发）；过刊回溯是学术期刊的重要工作，在适当时机，本刊将创刊以来所有文章上传至本刊网站，实现可检索和开放存取等等措施。

### 2.3 展望

十二年光阴荏苒，我们始终孜孜以求，从未懈怠。追忆往昔，《中国肺癌杂志》有目共睹的成长足迹鼓舞着我们；身在当下，Medline给予我们的肯定激励着我们；遥看前方，为把《中国肺癌杂志》专业化、国际化之路更深更远地走下去，未来光辉的前途指引着我们。《中国肺癌杂志》被Medline收录，是本刊发展历程上的一个里程碑，但这并不是终点，而是一个崭新的起点。我相信，《中国肺癌杂志》一定会随着中国肺癌基础、临床科研水平的进步，取得更大的成绩；中国广大肺癌工作者被国际同道所认可，成为国际肺癌领域的中坚力量；中国的肺癌事业也会跻身世界前列，造福全人类。

